# The use and abuse of genetic marker-based estimates of relatedness and inbreeding

**DOI:** 10.1002/ece3.1541

**Published:** 2015-07-14

**Authors:** Helen R Taylor

**Affiliations:** Allan Wilson Centre, School of Biological Sciences, Victoria University of WellingtonKelburn Parade, Wellington, New Zealand

**Keywords:** COANCESTRY, estimators, inbreeding, relatedness

## Abstract

Genetic marker-based estimators remain a popular tool for measuring relatedness (*r*_*xy*_) and inbreeding (*F*) coefficients at both the population and individual level. The performance of these estimators fluctuates with the number and variability of markers available, and the relatedness composition and demographic history of a population. Several methods are available to evaluate the reliability of the estimates of *r*_*xy*_ and *F*, some of which are implemented in the program COANCESTRY. I used the simulation module in COANCESTRY since assess the performance of marker-based estimators of *r*_*xy*_ and *F* in a species with very low genetic diversity, New Zealand’s little spotted kiwi (*Apteryx owenii*). I also conducted a review of published papers that have used COANCESTRY as its release to assess whether and how the reliability of the estimates of *r*_*xy*_ and *F* produced by genetic markers are being measured and reported in published studies. My simulation results show that even when the correlation between true (simulated) and estimated *r*_*xy*_ or *F* is relatively high (Pearson’s *r* = 0.66–0.72 and 0.81–0.85, respectively) the imprecision of the estimates renders them highly unreliable on an individual basis. The literature review demonstrates that the majority of studies do not report the reliability of marker-based estimates of *r*_*xy*_ and *F*. There is currently no standard practice for selecting the best estimator for a given data set or reporting an estimator’s performance. This could lead to experimental results being interpreted out of context and render the robustness of conclusions based on measures of *r*_*xy*_ and *F* debatable.

## Introduction

Quantifying the degree of relatedness between individuals within a population is a key to many genetic research topics (Ritland 1996; Lynch and Ritland 1999). Estimates of relatedness have been used widely in studies of gene flow (Morin et al. 1994; Streiff et al. 1999), kin selection and cooperative breeding (Peters et al. 1999; Hatchwell et al. 2014), trait heritability (Kruuk 2004), social behavior and structure (Ward 1983; Laidlaw and Page 1984; Queller et al. 1988), and to manage conservation breeding programs (Jones et al. 2002; Kozfkay et al. 2008; Goncalves da Silva et al. 2010; Bergner et al. 2014), while accurate estimates of individual inbreeding are pivotal to studies of inbreeding depression (e.g., Grueber et al. 2010; Nielsen et al. 2012). The coefficient of relatedness (*r*_xy_) measures the expected proportion of shared alleles between pairs of individuals that are identical by descent (IBD) (Blouin 2003), while an individual’s inbreeding coefficient (*F*) is the probability of IBD of two alleles at a locus in an individual (i.e., the probability they were inherited from a common ancestor) (Wright 1921; Malécot 1948). These metrics can be estimated at an individual level or averaged over populations. Pedigrees are often suggested as the best method for estimating *r*_xy_ and *F* (Pemberton 2008; Santure et al. 2010), but this method is problematic for three reasons. First, pedigrees assume unrelated founders, which is rarely the case and can lead to the underestimation of *r*_xy_ and *F* (Jones et al. 2002; Russello and Amato 2004). Second, pedigree-based estimates of *r*_xy_ and *F* are unable to account for the variance in IBD that occurs by chance between dyads or individuals with the same pedigree-based *r*_xy_ or *F,* respectively (Hill and Weir 2011). Finally, the data required for pedigree construction is often lacking for wild populations, especially those that have not been monitored long term, and inaccurate pedigrees will lead to inaccurate estimates of *r*_xy_ and *F* (Pemberton 2008; Jones and Wang 2009).

The coefficients of relatedness and inbreeding can also be estimated directly from genetic markers. This is not a new concept (Morton et al. 1971), but has become more popular with the increasing availability of relatively large panels of microsatellite and, more recently, single nucleotide polymorphism (SNP) markers. Seven widely used relatedness estimators have been developed since the late 1980s. These can be divided into two types: moment estimators (that estimate the relatedness between individuals in terms of probabilities of identity by descent) (Queller and Goodnight 1989; Li et al. 1993; Ritland 1996; Lynch and Ritland 1999; Wang 2002) and likelihood methods (that calculate the probability of individuals falling into a particular relationship given the marker data available) (Anderson and Weir 2007; Wang 2007). It is also possible to calculate *F* for individuals using the Ritland (1996) or Lynch and Ritland (1999) moment estimators, or the Anderson and Weir (2007) or Wang (2007) likelihood estimators (Wang 2011). The performance of marker-based estimators of *r*_xy_ and *F* has been shown to be affected by the relatedness composition of the population in question (Van de Casteele et al. 2001; Csilléry et al. 2006), the number and polymorphism of loci used (Blouin 2003) and the demographic history of a population (Robinson et al. 2013). No one estimator performs best in all scenarios and it is recommended that simulations are conducted to select the most appropriate estimator for a given scenario (Van de Casteele et al. 2001; Wang 2011).

In studies relying on marker-based estimates of *r*_*xy*_ and/or *F* to make inferences regarding biological systems, conducting a priori simulations is important for two reasons: (1) selecting the correct estimator(s) for use with a given marker set, and (2) assessing the likely reliability of any estimates generated. Prior knowledge of these two factors is essential for evaluating the robustness of any conclusions based on marker-based estimates of *r*_*xy*_ or *F*. This is particularly important for species of conservation concern, which often show low genetic diversity as a result of population bottlenecks, rendering markers less informative for estimating *r*_*xy*_ and *F*. Where high-density SNP panels or large numbers of microsatellite markers are not available for species with low genetic diversity, marker-based estimates of *r*_*xy*_ or *F* may be highly biased and/or imprecise, rendering any conclusions based on these estimates potentially unsound (see Van Horn et al. 2008 for an illustration of this). The importance of a priori simulations for studies using marker-based estimators of *r*_*xy*_ and *F* has already been stated several times in the scientific literature (Van de Casteele et al. 2001; Wang 2011; Pew et al. 2014). The program COANCESTRY (Wang 2011) estimates *r*_*xy*_ and *F* using both moment and likelihood estimators, but also facilitates a priori simulations to assess estimator performance and aid the section of the best estimator for a given data set. The release of this program might be expected to have aided the broad adoption of a priori evaluation of estimators (particularly in studies using COANCESTRY) to ensure that conclusions based on direct marker-based estimates are always as robust as possible. This is of particular concern in cases where recommendations for species management actions are based on such conclusions. However, it is unclear whether these recommendations have been heeded by scientists implementing marker-based estimates of *r*_*xy*_ and *F*.

This study has two aims: (1) to illustrate the necessity of using a priori simulations to thoroughly evaluate performance of marker-based estimators of *r*_*xy*_ and *F* at a population and individual level, and (2) to quantify how often a priori simulations are used to assess estimator performance and select the best estimator of *r*_*xy*_ and *F* in scientific studies. As such, this study is divided into two sections. First, I conduct simulations using empirical allele frequencies to select the best estimator and evaluate the performance of marker-based estimators of *r*_*xy*_ and *F* in a species with very low genetic diversity, New Zealand’s little spotted kiwi (*Apteryx owenii*) (LSK). Second, I review the scientific literature for studies that have used COANCESTRY to estimate *r*_*xy*_ and/or *F* and ask whether and how researchers select specific estimators and evaluate the power of the genetic marker sets available to them. Specifically I ask: (1) Can accurate estimates of *r*_*xy*_ and/or *F* be generated via marker-based estimators for species with very low genetic diversity, such as LSK? (2) How are marker-based estimators of *r*_*xy*_ and *F* selected by the studies that use them? (3) Is the likely accuracy of marker-based estimates of *r*_*xy*_ and/or *F* assessed by the majority of studies employing them?

## Methods

### Marker-based relatedness and inbreeding simulations

#### Study species

Little spotted kiwi are a flightless, nocturnal ratite endemic to New Zealand. Although once widespread throughout New Zealand, all mainland LSK populations had been extirpated by introduced predators by the late 1980s. The species survived solely due to a successful population on Kapiti Island that was founded by, at most, five birds in 1912 (Ramstad et al. 2013). Between 1982 and 2010, individuals from Kapiti Island were translocated to found new LSK populations on six other islands and in one mainland island sanctuary, leading to a current population of ∼1700 birds (H. Robertson, unpubl. data.). The new populations were founded with between two and 40 individuals and these secondary bottlenecks, combined with the original bottleneck of ≤5, have left LSK with very low genetic diversity and an elevated risk of inbreeding (Ramstad et al. 2013). Thus, measuring *r*_xy_ and *F* within LSK populations is of interest to the future protection and management of this species, but is rendered challenging due to a lack of pedigree information for any LSK population. As there are currently 21 polymorphic microsatellite markers characterized for LSK and two of the extant populations (those on Long Island and in Zealandia ecosanctuary) have been extensively sampled for DNA, marker-based estimates of *r*_xy_ and *F* represent a potentially useful tool for the management of this species.

#### Simulations

Simulations in the program COANCESTRY were used to select the best estimator from the seven implemented in the program and to assess the performance of this best estimator using empirical allele frequencies across 21 microsatellite markers from the Long Island and Zealandia populations of LSK. Simulated populations for relatedness testing consisted of 600 dyads spread equally across six categories of relatedness: parent–offspring (*r*_xy_ = 0.5), full siblings (*r*_xy_ = 0.5), half siblings/avuncular/grandparent–grandchild (*r*_xy_ = 0.25), first cousins (*r*_xy_ = 0.125), second cousins (*r*_xy_ = 0.03125), and unrelated (*r*_xy_ = 0). For inbreeding coefficients, the simulated data set consisted of 2100 individuals with inbreeding coefficients that varied from 0 to 1 at intervals of 0.05, and 100 individuals in each inbreeding category. Both approaches were modeled after those taken by Brekke et al. (2010). In both relatedness and inbreeding simulations, the allele frequencies, missing data and error rates for simulated microsatellite loci genotypes were based on those found in two different LSK populations: Long Island and Zealandia (Table S1). These populations were selected because they were founded with the lowest (two) and highest (40) numbers of individuals and thus represent the minimum and maximum amount of genetic diversity for any recently translocated LSK populations. Both populations have also been subject to extensive genetic sampling, with genotypes available for 43 Long Island and 113 Zealandia birds (86 and 94% of the current estimated population sizes, respectively).

Estimates produced by the triadic likelihood (TrioML) method were the most closely correlated with the simulated true relatedness and inbreeding coefficients for both the Long Island and Zealandia marker sets. Thus, estimates from the other six estimators were excluded from further analysis. Wilcoxon sign rank tests were used to test for significant differences between simulated and TrioML-estimated relatedness values, and the coefficient of variation (Abdi 2010) was calculated for estimates of each category of relatedness. Linear regression of simulated inbreeding coefficients against TrioML estimates of inbreeding coefficients was conducted to assess the bias and precision of estimates of *F*. All statistical analyses were conducted in R (R Development Core Team 2013).

### Literature review

A Web of Science search was conducted in September 2014 for all papers that had cited COANCESTRY since its publication in 2011. Marker-based estimators have been in use for several decades prior to the release of COANCESTRY, but COANCESTRY facilitates simulation-based evaluation of the discriminatory power of the markers being used. Thus, it was of interest to quantify which studies using COANCESTRY to estimate relatedness or inbreeding had also used it to select the best estimator and determine the likely performance of that estimator for their data set. This search resulted in a total of 82 peer-reviewed publications, seven of which were species specific or wider topic reviews, or theoretical papers and were thus removed from further analysis. The remaining 75 papers were analysed to determine the purpose of each study and the metrics being estimated, the type and number of molecular markers used to estimate the relevant metrics, and the use of simulations to select appropriate estimators and assess their reliability. Papers are not always listed on Web of Science immediately on publication. Thus, it is acknowledged that some recent studies using COANCESTRY may have been omitted from this review (e.g., Bergner et al.’s study on relatedness in kākāpō (*Strigops habroptilus*) (2014)). A full list of all the studies reviewed can be found in the Supplementary Information for this paper (Table S2).

## Results

### Marker-based relatedness and inbreeding simulations

**Figure 1:**
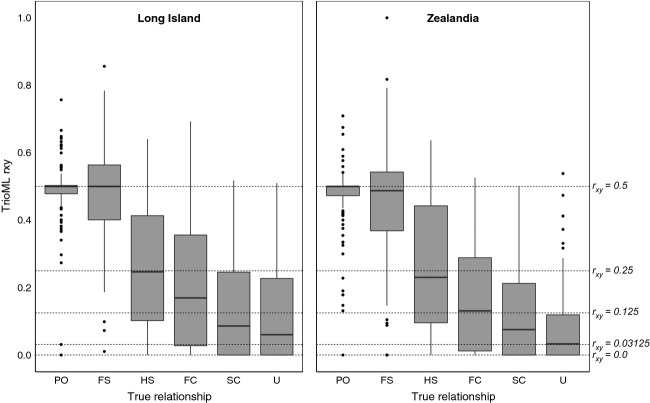
Spread of relatedness coefficients estimated by TrioML in COANCESTRY for simulated dyads in six true relationship categories using simulated genotypes based on the Long Island and Zealandia microsatellite marker sets. Boxes represent the upper and lower quartiles, divided by the median. The 10 and 90 percent quartiles are depicted by lines and dots represent the outliers. Dashed horizontal lines mark true *r*_xy_ coefficients of 0.5 (parents-offspring (PO) and full siblings (FS)), 0.25 (half siblings/avuncular/grandparent-grandchild (HS)), 0.125 (first cousin (FC)), 0.01325 (second cousin (SC)) and 0 (unrelated (U)).

**Figure 2:**
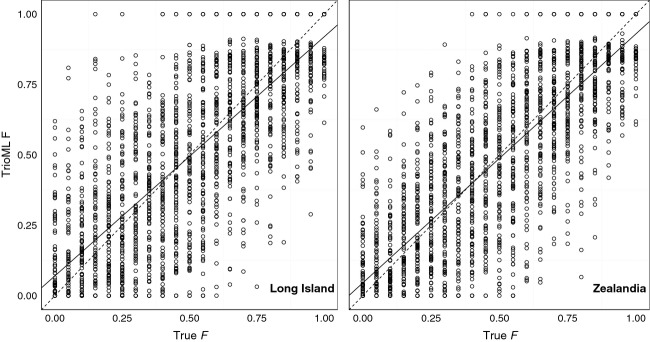
Regression line (solid) versus 1:1 line (dashed) for regressions of inbreeding coefficients estimated using TrioML in COANCESTRY versus true inbreeding coefficients for simulated individuals with genotypes based on the Long Island and Zealandia microsatellite marker sets. Long Island *β *= 0.85, *r*^2^ = 0.61, *F* = 3260, *P* < 0.001. Zealandia *β *= 0.88, *r*^2^ = 0.67, *F* = 4257, *P* < 0.001.

In general, marker-based estimators of both coefficients performed worse when using the empirical allele frequencies available for Long Island than those for Zealandia (Table1, Figs.1 and 2). However, even in the more variable Zealandia population, estimates of *r*_*xy*_ and *F* ranged widely, particularly for distantly related individuals and those that were in the middle range of inbreeding coefficients simulated (Figs.1 and 2).

**Table 1:** Differences between relatedness coefficients estimated using TrioML in COANCESTRY and true *r*_xy_ for simulated dyads in six relationship categories. Simulated genotypes of dyads were based on the Long Island and Zealandia microsatellite marker sets

Allele frequencies used	True relationship	Actual *r*_xy_	TrioML mean estimated *r*_xy_ (±95% CIs)	Wilcoxon *V*	*P*	Coefficient of variation
Long Island	Parent–offspring	0.5	0.48 (±0.02)	1059	NS	20%
	Full siblings	0.5	0.48 (±0.03)	2116	NS	31%
	Half siblings	0.25	0.25 (±0.04)	2412	NS	70%
	First cousins/avuncular	0.125	0.21 (±0.04)	1603	***	89%
	Second cousins	0.01325	0.14 (±0.03)	1028	***	117%
	Unrelated	0	0.15 (±0.04)	1891	***	124%
Zealandia	Parent-offspring	0.5	0.47 (±0.02)	914	**	23%
	Full siblings	0.5	0.46 (±0.03)	2371	*	37%
	Half siblings	0.25	0.25 (±0.04)	2490	NS	72%
	First cousins/avuncular	0.125	0.16 (±0.03)	2145	NS	98%
	Second cousins	0.01325	0.13 (±0.03)	1031	***	115%
	Unrelated	0	0.08 (±0.02)	2145	***	144%

* = *P* < 0.05.

** = *P* < 0.01.

*** = *P* < 0.001. NS = Not significant.

COANCESTRY reported Pearson’s correlation coefficients of 0.66 and 0.72 between TrioML-estimated and simulated values of *r*_xy_ for Long Island and Zealandia, respectively. In spite of these relatively strong correlations, estimates of *r*_xy_ ranged widely in all six categories of kinship tested (Fig.1 and Table1). For the Long Island simulations, mean estimates of *r*_xy_ were not significantly different from simulated values for parent–offspring, full-sibling and half-sibling dyads, but were significantly different for first cousin, second cousin, and unrelated dyads (Table1). When the Zealandia allele frequencies were used in simulations, mean *r*_xy_ estimates for half sibling dyads and first cousin dyads were not significantly different from the simulated values, but those for all four other categories were (Table1). For both populations, the coefficient of variation (CV) of estimates increased with decreasing simulated *r*_xy_ (Table1). CV ranged from 20% (Long Island) and 23% (Zealandia) for parent–offspring dyads to 124% (Long Island) and 144% (Zealandia) for unrelated dyads.

Pearson’s correlation coefficients for TrioML-estimated and simulated values of *F* were 0.81 for Long Island and 0.85 for Zealandia. Again, in each case, TrioML estimates of inbreeding coefficients varied widely for a given true value of *F* (Fig.2). This variation was greatest between simulated *F* values of ∼0.4–0.65, regardless of which population’s allele frequencies were used, with less variation in estimates of *F* for individuals with very low (0–0.1) or very high (0.9–1.0) *F*. Linear regression analyses indicated a slight bias in estimates of *F* for both populations (Long Island *β *= 0.85; Zealandia *β *= 0.88) with marker-based measures tending to overestimate *F* when simulated *F* was low and underestimate *F* when it was high (Fig.2). Zero bias only occurred at simulated *F* of ∼0.4–0.5 (Fig.2). Linear regression also illustrated a lack of precision of marker generated estimates of *F* for both populations. Although precision was higher for the Zealandia population (*r*^2^* *=* *0.67) than the Long Island population (*r*^2^* *=* *0.61), neither set of estimates were especially precise (Fig.2).

### Literature review

A total of 75 papers citing COANCESTRY were reviewed for this study (Table S2). These papers spanned 33 different peer-reviewed journals and included studies on species of mammals (41%), birds (19%), fish (9%), insects (9%), reptiles (8%), plants (8%), gastropods (1%), amphibians (1%), maxillopods (1%), and malacostracans (1%). The majority (77%) of papers that have cited COANCESTRY to date have been solely concerned with estimating *r*_xy_, with 12% of studies focussed on estimating just *F*, and 9% on a combination of the two metrics. Two of the papers used COANCESTRY to check for identical individuals in a sample or to simulate a set of individual genotypes rather than estimating *r*_xy_ or *F* and were discarded from further analysis, leaving 73 papers. A total of 23% of the papers reviewed estimated *r*_xy_ of dyads and/or *F* of individuals, 53% estimated population means, and 23% estimated both.

The purpose of estimating *r*_xy_ and/or *F* varied widely from study to study. Only three papers (4%) used marker-based estimates to detect inbreeding depression. Other uses included assessing social organization, excluding related individuals from downstream analyses, investigating co-operative breeding, detecting sibling-related cannibalism, and facilitating the development of SNP panels. Microsatellite markers were the most commonly used tool for estimating *r*_xy_ and/or *F* (96% of studies), with anywhere from 4 to 33 markers implemented.

The vast majority (95%) of studies reported which of the seven available estimators was used. However, only 28% of the studies that reported the estimator justified their choice in any respect and even fewer (14%) used a simulation-based approach to select the appropriate estimator for the markers used in the study. Of those that did conduct simulations to select an estimator, 70% reported the performance of their chosen estimator in some form. This means that of 73 studies estimating relatedness and/or inbreeding directly using genetic markers, 9% (seven papers) tested and reported the performance of the implemented estimator. These seven studies variously used Pearson’s *r* (four studies), the raw variance of estimates (one study), the *r*^2^ of estimates (one study), and statistical power (PW_*R*_) calculated in the program KinInfor (Wang 2006) (one study). Of the 22 studies that estimated individual *r*_*xy*_ and/or *F* (alongside or instead of population means), 91% specified the estimator used. Of these, 32% justified their choice of estimator, 19% used a simulation-based approach to select the best estimator for the marker set available, and 13% (four papers) reported the performance of their chosen estimator.

## Discussion

The simulation results presented here underline the importance of conducting a priori tests of estimator accuracy for a given marker set before estimating *r*_*xy*_ and *F*. They also illustrate that, even when relatively strong correlations between true and estimated values of *r*_*xy*_ and *F* are predicted, individual estimates can still vary widely for a given value, causing precision to be low. In light of this, it is troubling that so many papers reviewed here failed to investigate and report the performance of their selected estimator of *r*_*xy*_ or *F*. Unless standard practice for selecting and assessing the performance of marker-based estimators of *r*_*xy*_ and *F* is implemented, the conclusions based on such estimates remain open to debate and should be treated with caution.

### Using marker-based estimates of *r*_xy_ and *F* in little spotted kiwi

In the relatedness simulation tests using empirical allele frequencies and the existing LSK microsatellite marker set, the precision of estimates decreased in tandem with relatedness. This is likely due to the increase in variance of IBD between dyads with the same *r*_xy_ as relatedness decreases. It is thought that, on average, more than twice as many loci are required to precisely discriminate second degree relatives from unrelated dyads than first degree (Blouin 2003). Variance in IBD is likely also the cause of the higher precision seen here for parent–offspring dyads versus full-sibling dyads. Although average *r*_xy_ for both kinds of dyads is 0.5, the pattern of allele sharing is different. Offspring will almost always inherit 50% of their alleles from each parent whereas full siblings will share 50% of their alleles on average, but with more variance from dyad to dyad (Weir et al. 2006).

All marker-based estimators measure *r*_*xy*_ and/or *F* relative to a reference population, which is assumed to contain noninbred and unrelated individuals. In the majority of studies (as here), the current population also serves as the reference, resulting in relatedness being estimated relative to all individuals in the sample rather than to an separate unrelated sample (Wang 2014). When marker diversity is low, there will be little difference in the genetic similarity of unrelated and highly related individuals due to increased identity by state (IBS). Thus, for both populations, estimates of *r*_*xy*_ were (on average) underestimates of closely related dyads, overestimates of loosely or unrelated dyads and there was a roughly equal amount of under and overestimation for dyads with a true *r*_xy_ of 0.25, which is halfway between the minimum and maximum level of relatedness in the simulated population. A similar pattern was seen in the bias of individual inbreeding estimates.

The microsatellite markers currently available for LSK have low power to directly estimate pairwise relatedness or individual inbreeding, even in Zealandia, which has some of the highest allelic diversity of any LSK population (Taylor 2014). The relatively high allelic diversity in the Zealandia population resulted in a closer overall correlation between estimated and simulated values of pairwise relatedness and individual inbreeding than that seen for Long Island. The overall bias of relatedness and inbreeding estimates was not severe for either marker set, but the variability in estimates of either metric would render estimates highly unreliable at an individual level, especially with the relatively small sample sizes available for this and many other threatened species. Marker-based estimates of relatedness and inbreeding are expected to be more robust when averaged over large numbers of individuals (Rollins et al. 2012); thus, these estimators could potentially be used to generate mean values of *r*_*xy*_ and *F* for LSK populations, but not for dyads or individuals. Conclusions regarding inbreeding depression using individual estimates generated in this fashion would be highly questionable. It would also be unadvisable to use such estimates to select individuals for use in translocation-based management. The poor performance of the estimators in LSK at an individual level is not immediately apparent when viewing COANCESTRY generated Pearson’s *r* statistic in isolation. This highlights the need for a more comprehensive assessment of estimator performance, especially when the intention is to estimate *r*_*xy*_ for individual dyads or *F* for individuals.

The low reliability of marker-based estimates of *r*_*xy*_ and *F* at the individual level in LSK is unfortunate as there is currently no pedigree information for any population of this species. As the extant population of LSK is descended from, at most, five birds (Ramstad et al. 2013) and all eight subpopulations within this species were founded with between two and 40 individuals, inbreeding depression and the selection of individuals for future translocations are topics of interest for ongoing management of LSK. However, the lack of pedigree data and extremely low genetic variation seen in LSK will render attempts to accurately quantify *r*_*xy*_ or *F* in this species challenging. The scenario exhibited by LSK is not uncommon, with many wild populations lacking pedigree data (Pemberton 2008) and many threatened species exhibiting low genetic variation (e.g., Haig and Avise 1996; Leonard et al. 2005; Schultz et al. 2009; Miller et al. 2011; Chen et al. 2012). Clearly, new tools are required to tackle the issues of estimating *r*_*xy*_ and *F* in such species; high-density SNP panels (e.g., Santure et al. 2010; Saura et al. 2013) and runs of homozygosity (e.g., McQuillan et al. 2008; Purfield et al. 2012; Prado-Martinez et al. 2013) are currently the most promising new techniques, and these will become more feasible for nonmodel threatened species as the cost of next-generation sequencing continues to fall. However, as the review conducted here illustrates, microsatellites are still the prevalent molecular tool in use for estimating *r*_*xy*_ and *F*, and assessing and reporting the power of these marker sets for estimating *r*_*xy*_ and *F* remains an important issue.

### Current uses and reliability assessments for marker-based estimators of *r*_xy_ and *F*

Marker-based estimates of *r*_*xy*_ and *F* are in use across a variety of study areas in a diverse array of taxa. The results from the COANCESTRY-based literature review show that, currently, genetic markers are more often used to estimate *r*_*xy*_ than *F*. This is possibly due to the fact that COANCESTERY is promoted as a relatedness estimation tool, with inbreeding estimates as an added bonus, and the fact that pedigree analysis is still widely encouraged as the best way of estimating *F*. As a result, marker-based estimates of *F* from COANCESTRY are not currently used extensively to detect inbreeding depression, but COANCESTRY is used heavily in behavioral studies to estimate *r*_*xy*_.

The results from the LSK simulation experiment show the importance of proper estimator selection and a priori assessment of estimator performance in more depth than that currently facilitated by COANCESTRY. However, the literature review data illustrate that these procedures are not being followed or their results not being reported in the majority of studies using molecular markers to estimate *r*_*xy*_ and *F*. In some cases, the performance of the estimator was reported, but found to be low, and this was not discussed in terms of the validity of the conclusions formed (e.g., Hammerly et al. 2013). The fact that studies involving marker-based estimates of *r*_*xy*_ and *F* are sometimes published without stating the estimator used is surprising as it reduces the repeatability of the study. When estimators are reported, the methods of justifying their selection are wide ranging. Only 10 of the 73 studies reviewed stated that the estimator used was selected due to it outperforming the other available estimators in simulation tests. Other studies went as far as to research the best estimator based on previous studies or made generic statements regarding the performance of the estimator in different circumstances, but did not assess that estimator based on their own markers – a potentially critical error.

When estimator performance was reported, the methods of doing so also varied, with five measures employed across seven studies. Rollins et al. (2012) used a combination of Pearson’s *r* correlation between COANCESTRY and pedigree estimates to select the best estimator and PW_*R*_ calculated in the program KinInfor (Wang 2006) to quantify the power of their marker set. This was one of the most comprehensive evaluation procedures undertaken in any of the studies reviewed and could potentially form the basis for a standardized protocol for estimator evaluation and selection. Vangestel et al. (2011) used simulations and Pearson’s *r* to select the best estimator for *r*_*xy*_ and feature a figure similar to Figure1 illustrating the variation in their simulation estimates. Bonin et al. (2012) used the variance of different estimators in simulations to select the appropriate method. There is currently no apparent agreed upon best practice for reporting estimator performance and this reduces the comparability of studies. A standard measure of marker power/reliability of estimates should be adopted – even if it is the potentially misleading Pearson’s *r* correlation, but a thorough approach such as that of Rollins et al. (2012) would be preferable. As illustrated by the LSK example presented here, metrics that encompass variation in estimates at an individual level are particularly important for studies attempting to estimate *r*_*xy*_ and *F* for dyads and individuals, respectively. The program KinInfor offers several metrics for assessing the informativeness of a given marker set and these, in tandem with a simulation approach and examination of correlations between true and estimated values plus the variance of estimates could provide a more reliable and repeatable approach. More recently, an R implementation of COANCESTRY called *related* has been developed (Pew et al. 2014). This package not only retains the original simulation functions of CONACETRY, but also outputs boxplots comparing the performance of four commonly used *r*_*xy*_ estimators (Queller and Goodnight 1989; Li et al. 1993; Lynch and Ritland 1999; Wang 2002) across relatedness values. This is designed to make it even easier for researchers to reliably assess likely estimator performance and select the optimum estimator for their chosen data set.

Even with large numbers of markers, marker-based estimators are more suitable for calculating population-wide mean estimates of *r*_*xy*_ than for individual dyads (Santure et al. 2010). Indeed, pairwise relatedness estimators were never intended to classify pairs of individuals into discrete categories (Csilléry et al. 2006). In light of this, it is reassuring that the majority of studies reviewed here only use COANCESTRY to estimate mean values of *r*_*xy*_ and *F* across groups of individuals. This certainly does not negate the need to assess marker power and reliability of estimates, but it means that, in general, the mean estimates presented in these studies should be more reliable than those for dyads or individuals. However, within the 34 papers that did estimate *r*_*xy*_ and *F* on an individual basis, only four reported the power of the marker set or likely reliability of the estimates used, placing the conclusions of the remaining papers in doubt, especially given that some of these papers used as few as 7–9 microsatellites to generate their estimates.

A final issue addressed in only one of the reviewed papers (Domingos et al. 2014) is that of the reference population used when estimating relatedness using genetic markers (Wang 2014). As the estimator can only calculate relatedness based on the individuals available, the resulting estimates are relative to the reference population. Thus, if an unrelated reference population is used in *r*_*xy*_ estimation (as in Domingos et al.), then the results will be far closer to reality than if, as is often the case (as here in the LSK example), the current sample also acts as the reference (Wang 2014). This is analogous to the issue of assuming pedigree founders are unrelated when calculating pedigree inbreeding coefficients – it is usually untrue and will likely lead to underestimation of inbreeding (Jones et al. 2002; Russello and Amato 2004). If true relatedness in the reference population is high, then marker-based estimates of *r*_*xy*_ will be downwardly biased; marker-based estimates are relative rather than absolute measures and this should be acknowledged in the studies that use them.

## Conclusion

Little spotted kiwi are an excellent example for demonstrating that microsatellite markers are not always sufficient to reliably estimate *r*_*xy*_ and *F* at an individual level in threatened species with low genetic diversity. High-density SNP panels and whole genome sequencing will go some way to addressing the issues of reliability in marker-based estimators, but even large panels of SNPs cannot always reliably estimate *r*_*xy*_ or *F* on an individual basis (Santure et al. 2010). The review conducted here illustrates that, currently, microsatellite markers remain the dominant tool for estimating relatedness and inbreeding. This is particularly true in conservation studies where funds are often scant and behavioral studies where genetics is not usually the main focus of the study. In both these cases, it is still more prudent financially to use an existing microsatellite marker set rather than invest in a SNP discovery process.

The use of simulation-based approaches to select the correct estimator for *r*_*xy*_ and *F* and to assess the likely reliability of that estimator given the available marker set has been recommended repeatedly (Van de Casteele et al. 2001; Wang 2011). Several papers have demonstrated useful simulation approaches for assessing the power of a marker set to estimate *r*_*xy*_ and *F* (Rollins et al. 2012; Robinson et al. 2013) and software now exists that facilitates such exploration for naïve users. In spite of this, results from marker-based *r*_*xy*_ and *F* estimators are regularly being used to form conclusions and recommendations without the reliability of these estimates being assessed. When they are assessed, it is often via a simple correlation metric which, as the LSK example presented here shows, can be misleading as to the reliability of estimates on an individual basis. With the absence of pedigrees for many wild populations, marker-based estimates are likely to remain popular and have the potential to be useful, especially as more markers become available. However, in order for the studies employing marker-based estimators to draw robust conclusions that withstand close scrutiny, a standard practice for evaluating these techniques must be implemented and adhered to.

## Supporting Information

**Table S1.**Locus information entered into COANCESTRY for the relatedness and inbreeding coefficient simulations. **Table S2.** Summary of literature review of papers citing COANCESTRY for use in estimating relatedness or inbreeding coefficients.
