# Supplementary material for: The use and abuse of genetic marker-based estimates of relatedness and inbreeding
Source: Ecol Evol. 2015 Jul 14;5(15):3140–50. doi: 10.1002/ece3.1541 (PMC4559056; doi:10.1002/ece3.1541)
Supplement: Supplementary file 1 [file ece30005-3140-sd1.docx]

Table S1. Locus information entered into COANCESTRY for the relatedness and inbreeding coefficient simulations. All figures are based on real data for the Long Island and Zealandia LSK marker set. Dashes in the Long Island marker set indicate loci that were fixed in that population.

|  | Long Island | | | Zealandia | | |
| --- | --- | --- | --- | --- | --- | --- |
| Locus | Allele frequencies | Proportion missing data | Genotyping error | Allele frequencies | Proportion missing data | Genotyping error |
| Aptowe1 | 0.826, 0.174 | 0 | 0 | 0.004, 0.769, 0.227 | 0 | 0.07 |
| Aptowe2 | - | - | - | 0.103, 0.897 | 0 | 0.07 |
| Aptowe3 | 0.543, 0.457 | 0 | 0 | 0.862, 0.138 | 0.01 | 0 |
| Aptowe7 | - | - | - | 0.970, 0.030 | 0.01 | 0 |
| Aptowe8 | - | - | - | 0.021, 0.979 | 0 | 0 |
| Aptowe15 | 0.539, 0.461 | 0.16 | 0 | 0.624, 0.376 | 0.08 | 0 |
| Aptowe23 | 0.602, 0.398 | 0.05 | 0 | 0.129, 0.031, 0.317, 0.174, 0.335, 0.013 | 0.03 | 0 |
| Aptowe24 | - | - | - | 0.093, 0.907 | 0 | 0 |
| Aptowe28 | 0.778, 0.222 | 0.05 | 0.07 | 0.876, 0.124 | 0 | 0.07 |
| Aptowe29 | 0.205, 0.231, 0.564 | 0.09 | 0 | 0.178, 0.091, 0.630, 0.101 | 0.09 | 0 |
| Aptowe31 | 0.522, 0.478 | 0 | 0 | 0.585, 0.415 | 0.01 | 0 |
| Aptowe34 | - | - | - | 0.284, 0.716 | 0.04 | 0.07 |
| Aptowe35 | 0.261, 0.467, 0.272 | 0.02 | 0 | 0.017, 0.174, 0.709, 0.100 | 0 | 0 |
| Aptowe39 | 0.186, 0.814 | 0.02 | 0 | 0.570, 0.430 | 0 | 0.07 |
| Apt59 | 0.298, 0.702 | 0 | 0 | 0.563, 0.437 | 0.04 | 0 |
| Rowi2 | 0.786, 0.214 | 0.07 | 0 | 0.550, 0.405, 0.045 | 0.04 | 0 |
| Rowi12 | 0.293, 0.707 | 0 | 0 | 0.123, 0.877 | 0.02 | 0 |
| Rowi16 | 0.590, 0.410 | 0.09 | 0 | 0.318, 0.682 | 0.12 | 0 |
| Rowi23 | 0.250, 0.750 | 0.14 | 0 | 0.125, 0.875 | 0.26 | 0 |
| Rowi46 | 0.810, 0.190 | 0.05 | 0 | 0.767, 0.233 | 0.09 | 0 |
| Rowi69 | - | - | - | 0.442, 0.558 | 0.01 | 0 |

Table S2. Summary of literature review of papers citing COANCESTRY for use in estimating relatedness or inbreeding coefficients. Search was conducted based on all articles citing COANCESTRY according to Web of Science accessed on 22^nd^ January 2014. Any articles referring to COANCESTRY in a context other than to estimate relatedness or inbreeding (i.e. reviews or technical artciles) were discarded. *Msats = microsatellite markers, SNPs = single nucleotide polymorphisms. **Estimators are denoted as follows: 1=Queller and Goodnight (1989), 2=Li et al (1993), 3=Ritland (1996), 4=Lynch and Ritland (1999), 5=Wang (2002), 6=DyadML (Milligan 2003), 7=TrioML (Wang 2007), NS=Not stated.

| **Year** | **Authors** | **Journal** | **Volume and pages** | **Species** | **Marker*** | **# markers** | **Metric estimated** | **Population mean or individual** | **To detect inbreeding depression** | **Estimator used**** | **Justify estimator selection?** | **Simulations conducted?** | **Power reported?** | **Power** |
| --- | --- | --- | --- | --- | --- | --- | --- | --- | --- | --- | --- | --- | --- | --- |
| 2011 | Bodbyl-Roels and Kelly | Evolution | 65, 2541-2552 | *Mimulus guttatus* | Msats | 4 | *F* | Mean | No | 4 | No | No | No | NA |
| 2011 | King et al | Animal Behaviour | 82, 1337-1348 | Desert baboon (*Papio ursinus*) | Msats | 16 | *r*_xy_ | Individual | No | 7 | No | No | No | NA |
| 2011 | King et al | American Journal of Primatology | 73, 768-774 | Desert baboon (*Papio ursinus*) | Msats | 17 | *r*_xy_ | Individual | No | 7 | No | No | No | NA |
| 2011 | MacDougall-Shackleton et al | Conservation Genetics | 12, 1195-1203 | Song sparrow (*Melospiza melodia*) | Msats | 7 | *F* | Mean | No | 6 | No | No | No | NA |
| 2011 | Stenglein et al | Journal of Mammalogy | 92, 784-795 | Gray wolf (*Canis lupus*) | Msats | 9 | Both | Both | No | 7, 4, 1 | No | No | No | NA |
| 2011 | Vangestel et al | Molecular Ecology | 20, 4643-4653 | House sparrow (*Passer domesticus*) | Msats | 16 | *r*_xy_ | Both | No | 1 | Yes | Yes | Yes | r= 0.82, P< 0.001 |
| 2011 | Watts et al | Journal of Zoology | 285, 281-291 | Spotted hyena (*Crocuta crocuta*) | Msats | 10 | *F* | Mean | No | 3 | No | No | No | NA |
| 2012 | Bogadnowicz et al | Journal of Mammalogy | 93, 799-807 | Whiskered bats (*Myotis mystacinus*, *M.brandtii*, *M.alcathoe*) | Msats | 15 | *r*_xy_ | Mean | No | 7 | Yes | No | No | NA |
| 2012 | Bonin et al | Journal of Experimental Marine Biology and Ecology | 412, 13-19 | Antarctic fur seal (*Arctocephalus gazella*) | Msats | 18 | *r*_xy_ | Individual | No | 6 | Yes | Yes | Yes | s=0.001-0.013 |
| 2012 | Castagneyrol et al | PloS ONE | 7, e44287 | Oak (*Quercus robur*) | Msats | 12 | *r*_xy_ | Mean | No | 6 | Yes | Yes | No | NA |
| 2012 | Chernenko et al | Journal of Chemical Ecology | 38, 1474-1482 | Ant (*Formica fusa*) | Msats | 8 | *r*_xy_ | Mean | No | 1 | No | No | No | NA |
| 2012 | Dowling et al | Conservation Genetics | 13, 1073-1083 | Razorback sucker (*Xyrauchen texanus*) | Msats | 13 | *r*_xy_ | Mean | No | 7 | No | No | No | NA |
| 2012 | Dowling et al | Transactions of the American Fisheries Society | 141, 990-999 | Razorback sucker (*Xyrauchen texanus*) | Msats | 15 | *r*_xy_ | Mean | No | 7 | No | No | No | NA |
| 2012 | Guertin et al | The Journal of Wildlife Management | 76, 1540-1550 | River otter (*Lontra canadensis*) | Msats | 8 | *r*_xy_ | Mean | No | 1, 7 | No | No | No | NA |
| 2012 | Karamanlidis et al | European Journal of Wildlife Research | 58, 511-522 | Brown bear (*Ursus arctos*) | Msats | 10 | *r*_xy_ | Mean | No | 1, 7, 3 | No | No | No | NA |
| 2012 | Klepaker et al | Evolutionary Ecology Research | 14, 169-191 | Threespine stickleback (*Gasterosteus aculeatus* L.) | Msats | 23 | *r*_xy_ | Mean | No | 1 | No | No | No | NA |
| 2012 | Mattila et al | PNAS | 109, E2496-E2505 | Glanville fritillary butterfly (*Melitaea cinxia*) | Msats | 7 | Both | Mean | No | 6 | Yes | No | No | NA |
| 2012 | Rollins et al | Molecular Ecology | 21, 1727-1740 | Chestnut-crowned babler (*Pomatostomus ruficeps*) | Msats | 14 | *r*_xy_ | Mean | No | 1 | Yes | Yes | Yes | Power= 0.98 |
| 2012 | Stiver et al | Behavioural Ecology and Sociobiology | 66, 855-864 | Tessellated darter (*Etheostoma olmstedi*) | Msats | 11 | *r*_xy_ | Mean | No | 1 | No | No | No | NA |
| 2012 | Wikberg et al | PloS ONE | 7, e48740 | Black and white colobus monkey (*Colobuus vellerosus*) | Msats | 20 | *r*_xy_ | Mean | No | 6 | Yes | Yes | Yes | Spearman’s r=0.90, df=148, p<0.001 |
| 2013 | Ariani et al | Conservation Genetics | 14, 943-951 | Sand lizard (*Liolaemus lutzae*) | Msats | 7 | F | Mean | No | 7 | Yes | No | No | NA |
| 2013 | Bonin et al | Ecology and Evolution | 3, 3701-3712 | Antarctic fur seal (*Arctocephalus gazella*) | Msats | 17 | *r*_xy_ | Mean | No | 6 | No | No | No | NA |
| 2013 | Brante et al | PloS ONE | 8, e67050 | *Crepidula coquimbensis* | Msats | 5 | *r*_xy_ | Mean | No | 7 | No | No | No | NA |
| 2013 | Conson et al | Genetica | 141, 205-215 | *Luehea divaricata* | Msats | 9 | Both | Mean | No | 7 | No | No | No | NA |
| 2013 | Fitak et al | Conservation Genetics | 14, 1233-1241 | Mount Graham red squirell (*Tamiasciurus hudsonicus grahamensis*) | Msats | 8 | *r*_xy_ | Mean | No | 4 | No | No | No | NA |
| 2013 | Hammerly et al | Molecular Ecology | 21, 5313-5328 | Attwarter's prairie chicken (*Tympanuchus cupido attwateri*) | Msats | 5 | Both | Both | Yes | 7 | Yes | Yes | Yes | r=0.28, variance= 0.03 |
| 2013 | Harrison et al | Behavioural Ecology and Sociobiology | 67, 1915-1929 | White-browed sparrow weaver (*Plocepasser mahali*) | Msats | 13 | *r*_xy_ | Mean | No | NS | No | No | No | NA |
| 2013 | Harrison et al | Molecular Ecology | 22, 5700-5715 | White-browed sparrow weaver (*Plocepasser mahali*) | Msats | 10 | *r*_xy_ | Both | No | 1 | No | No | No | NA |
| 2013 | Herreman and Peacock | Ursus | 24, 148-163 | Polar bear (*Ursus maritimus*) | Msats | 20 | *r*_xy_ | Individual | No | 1 | No | No | No | NA |
| 2013 | Huchard et al | Molecular Ecology | 22, 4701-4086 | Grey mouse lemur (*Microcebus murinus*) | Msats | 13 | *r*_xy_ | Mean | No | 7 | No | No | No | NA |
| 2013 | Jonker et al | Molecular Ecology | 22, 5835-5847 | Barnacle Goose (*Branta leucopsis*) | SNPs | 384 | Both | Individual r, mean F | No | 6 for *F* NS for *r*_xy_ | No | No | No | NA |
| 2013 | Klauke et al | Molecular Ecology | 22, 2011-2027 | El oro parakeet (*Pyrrhua orcesi*) | Msats | 18 | Both | Mean | No | 4 | No | No | No | NA |
| 2013 | Kraus et al | Molecular Ecology | 22, 41-55 | Mallards (*Anas Platyrhynchos*) | SNPs | 384 | *r*_xy_ | Individual | No | 6 | Yes | Yes | No | NA |
| 2013 | Kurvers et al | Animal Behaviour | 86, 993-1001 | Barnacle Goose (*Branta leucopsis*) | SNPs | 374 | *r*_xy_ | Mean | No | 6 | Yes | Yes | Yes | r^2^=0.9 |
| 2013 | Lenz et al | Proc B | 280, 1762 | Galapagos sealion (*Zalophus wollebacki*) | Msats | 22 | *F* | Both | Yes | 7 | Yes | Yes | No | NA |
| 2013 | Lobo et al | Biotropica | 45, 185-194 | *Ceiba pentandra* | Msats | 7 | *r*_xy_ | Both | No | 7 | No | No | No | NA |
| 2013 | De Lorenzis et al | Molecular Biotechnology | 54, 634-642 | Aglianico grapevine (*Vitis vinifera* L.) | Msats | 21 | *r*_xy_ | Individual | No | NS | No | No | No | NA |
| 2013 | Lührs et al | Behavioural Ecology | 24, 21-28 | Fossa (*Cryptoprocta ferox*) | Msats | 16 | *r*_xy_ | Individual | No | 7 | Yes | No | No | NA |
| 2013 | Minhos et al | American Journal of Physical Anthroplogy | 150, 464-474 | Black and white & red colobus monkey (*Colobus polykonios, Procolobus bacchius teminckii*) | Msats | 15 | *r*_xy_ | Mean | No | 1 | No | No | No | NA |
| 2013 | Nomano et al | Animal Behaviour | 86, 277-289 | Chestnut-crowned babler (*Pomatostomus ruficeps*) | Msats | 14 | *r*_xy_ | Individual | No | NS | No | No | No | NA |
| 2013 | Norman et al | PloS ONE | 8, e81012 | Brown Bear (*Ursus arctos*) | SNPs | 87 | *r*_xy_ | Individual | No | 4 | No | No | No | NA |
| 2013 | Phillips et al | Molecular Ecology | 22, 2301-2312 | Hawksbill turtle (*Eretmochelys imbricata*) | Msats | 33 | *r*_xy_ | Mean | No | 1 | No | No | No | NA |
| 2013 | Preston et al | Molecular Ecology | 22, 5027-5039 | Rifleman (*Acanthisitta chloris*) | Msats | 30 | *r*_xy_ | Mean | No | 1 | No | No | No | NA |
| 2013 | Purcell and Chapuisat | Evolution | 67, 1169-1180 | Ant (*Formica selysi*) | Msats | 8-9 | *r*_xy_ | Mean | No | 1 | No | No | No | NA |
| 2013 | Simpson et al | Ecology and Evolution | 3, 614-628 | Red Squirrel (*Sciurus vulgaris*) | Msats | 15 | *F* | Both | Yes | 7 | Yes | No | No | NA |
| 2013 | Sindičić et al | Conservation Genetics | 14, 1009-1018 | Eurasian lynx (*Lynx lynx*) | Msats | 20 | *F* | Mean | No | 7 and 6 | No | No | No | NA |
| 2014 | Baranski et al | PloS ONE | 9, e85413 | Black tiger shrimp (*Penaeus monodon*) | SNPs | 4237 | *r*_xy_ | Individual | No | NS | No | No | No | NA |
| 2014 | Bilgmann et al | Marine ecology progress series | 500, 265-279 | Short-beaked common dolphins (*Delphinus delphis*) | Msats | 14 | *r*_xy_ | Individual | No | 1 | No | No | No | NA |
| 2014 | Caniglia et al | Journal of Mammalogy | 95, 41-59 | Gray wolf (*Canis lupus*) | Msats | 12 | *F* | Both | No | 4 | No | No | No | NA |
| 2014 | Chakraborty et al | Conservation Genetics | 15, 897–907 | Asian elephant (*Elephas maximus*) | Msats | 12 | *r*_xy_ | Mean | No | 1 | No | No | No | NA |
| 2014 | Chang et al | American Journal of Primatology | 76, 72-83 | Sichuan snub-nosed monkey (*Rhinopithexus roxellana*) | Msats | 15 | *r*_xy_ | Mean | No | 5 | No | No | No | NA |
| 2014 | Chuluunbat et al | Animal Genetics | 45, 550–558 | Bactrian camel (*Camelus bactrianus*) | Msats | 17 | *r*_xy_ | Individual | No | 7 | No | No | No | NA |
| 2014 | Domingos et al | Aquaculture | 424-425, 174-182 | Barramundi (*Lates calcarifer*) | Msats | 10-17 | *r*_xy_ | Mean | No | 1 | No | No | No | NA |
| 2014 | Dreier et al | Molecular Ecology | 23, 3384–3395 | Bumblebees (*Bombus terrestris*, *B. lapidarius*, *B. pascuorum*, *B. hortorum* and *B. ruderatus*) | Msats | 10-14 | *r*_xy_ | Both | No | 4 | No | No | No | NA |
| 2014 | Evans et al | Proc B | 280, 1772 | Chinook salmon (*Oncorhynchus tshawytscha*) | Msats | 9 | *r*_xy_ | Individual | No | 1 | No | No | No | NA |
| 2014 | Feist et al | Conservation Genetics | 15, 953–966 | Hellbender salamander (*Cryptobranchus alleganiensis*) | Msats | 15 | *r*_xy_ | Both | No | 7 | No | No | No | NA |
| 2014 | Gamero et al | Behavioural Ecology and Sociobiology | 68, 73-83 | White-breasted mesites (*Mesitornis variegata*) | Msats | 9 | *r*_xy_ | Individual | No | 1 | No | No | No | NA |
| 2014 | Jensen et al | Conservation Genetics | 15, 261-274 | Western painted turtle (*Chrysemys pictabellii*) | Msats | 10 | *r*_xy_ | Mean | No | 4 | No | No | No | NA |
| 2014 | Kramer et al | Behavioural Ecology and Sociobiology | 68, 41-53 | Ant (*Temnothorax longispinosus*) | Msats | 4 | *r*_xy_ | Mean | No | 1 | No | No | No | NA |
| 2014 | Liu et al | Biological Conservation | 171, 288–298 | Przewalski’s horse (*Equus ferus przewalskii*) | Msats | 10 | *F* | Mean | No | 7 | No | No | No | NA |
| 2014 | De Lorenzis et al | Scientia Horticulturae | 169, 189–198 | Grapevine (*V. vinifera* L.) | Msats | 33 | *r*_xy_ | Individual | No | 4 and 5 | No | No | No | NA |
| 2014 | MacDonald et al | Heredity | 112, 333-342 | Tammar wallabies (*Macropus eugenii*) | Msats | 11 | Both | Mean | No | 7 | Yes | No | No | NA |
| 2014 | Newby et al | Environmental Bioogy of Fish | 97, 1057–1065 | Spotted eagle ray (Aetobatus narinari) | Msats | 8 | *r*_xy_ | Mean | No | 1 | No | No | No | NA |
| 2014 | Nowak et al | Conservation Genetics | 15, 743–747 | Brown bear (Ursus arctos) | Msats | 13 | Neither | NA | No | NA | NA | NA | NA | NA |
| 2014 | Phillips et al | Journal of Experimental Marine Biology and Ecology | 455, 29–37 | Hawksbill turtle (*Eretmochelys imbricata*) | Msats | 32 | *r*_xy_ | Individual | No | 1 | No | No | No | NA |
| 2014 | Pinho et al | PloS ONE | 9, e92507 | Lowland tapir (*Tapirus terrestris*) | Msats | 5 | *r*_xy_ | NA | No | NA | NA | NA | NA | NA |
| 2014 | Plough et al | BMC Evolutionary Biology | 14, 81 | Pacific gooseneck barnacle (*Pollicipes elegans*) | Msats | 3-5 | *r*_xy_ | Both | No | 2 | Yes | No | No | NA |
| 2014 | Puechmaille et al | PloS ONE | 9, e103452 | Mehely’s horseshoe bat (*Rhinolophus mehelyi*) | Msats | 18 | *r*_xy_ | Individual | No | 2, 5, 6 and 7 | No | No | No | NA |
| 2014 | Sardell et al | Molecular Ecology | 23, 933-946 | Lance-tailed manakins (*Chiroxiphia lanceolata*) | Msats | 20 | *r*_xy_ | Both | No | 1 | No | No | No | NA |
| 2014 | Schofield et al | Conservation Genetics | 15, 177-185 | Pygmy bluetongue lizard (*Tiliqua adelaidensis*) | Msats | 15 | *r*_xy_ | Both | No | 5 | No | No | No | NA |
| 2014 | Schuttler et al | PloS ONE | 9, e88074 | African forest elephants (*Loxodonta cyclotis*) | Msats | 12 | *r*_xy_ | Both | No | 1 | Yes | Yes | Yes | r= 0.911 |
| 2014 | Solazzo et al | Journal of Insect Coservation | 18, 69-75 | Ant (*Myrmica rubra*) | Msats | 5 | *r*_xy_ | Mean | No | 7 | Yes | No | No | NA |
| 2014 | Vanhala et al | Conservation Genetics | 15, 853–868 | Wood ant (*Formica aquilonia*) | Msats | 10 | *r*_xy_ | Both | No | 7 | Yes | No | No | NA |
| 2014 | While et al | Molecular Ecology | 23, 721-732 | Whites' skink (*Egernia whitii*) | Msats | 12 | *r*_xy_ | Mean | No | 1 | No | No | No | NA |
| 2014 | Wikberg et al | American Journal of Physical Anthroplogy | 153, 365-376 | Black and white colobus monkeys (*Colobus vellerosus*) | Msats | 17 | *r*_xy_ | Both | No | 1 | No | No | No | NA |
